# Evaluating the Role of Allopurinol in Mitigating Contrast-Induced Nephropathy in Percutaneous Coronary Intervention Patients: A Systematic Review and Meta-Analysis

**DOI:** 10.7759/cureus.76374

**Published:** 2024-12-25

**Authors:** Hassan Badreldin, Prabhakar Mallya Prabhakar

**Affiliations:** 1 Cardiology, Sheikh Khalifa Speciality Hospital, Ras Al Khaimah, ARE; 2 Diabetes and Endocrinology, Prabhath Diabetes Care Centre, Udupi, IND

**Keywords:** acute coronary syndrome, allopurinol, contrast-induced nephropathy, contrast media toxicity, meta-analysis, oxidative stress, percutaneous coronary intervention, renal protection, systematic review, xanthine oxidase inhibition

## Abstract

This meta-analysis investigates the potential of allopurinol to prevent contrast-induced nephropathy (CIN), a common and serious complication of percutaneous coronary intervention (PCI). CIN is particularly prevalent among high-risk populations, including patients with chronic kidney disease (CKD) or acute coronary syndrome (ACS), where the administration of contrast agents can exacerbate renal injury. Allopurinol, a xanthine oxidase inhibitor, is known for its dual action in reducing oxidative stress and uric acid production, positioning it as a promising therapeutic candidate to mitigate CIN. The analysis included eight studies encompassing a total of 929 patients undergoing PCI, with a mean age of 63 years. These studies compared the effects of allopurinol with placebo across varying doses and contrast agent types. The results demonstrated a significant reduction in CIN incidence with allopurinol, yielding a pooled odds ratio (OR) of 0.26 [95% CI (0.12, 0.56), P = 0.0006]. Despite the encouraging findings, moderate heterogeneity was observed (I² = 59%), likely arising from variations in study design, patient demographics, and the types of contrast agents used. Sensitivity analysis focusing on studies employing the contrast agent Omnipaque provided further support for the efficacy of allopurinol, with pronounced benefits observed in patients with CKD or ACS. These findings underline the potential of allopurinol as a preventive measure against CIN, especially in high-risk populations. However, the identified heterogeneity and inherent limitations of the included studies highlight the critical need for larger, well-designed randomized controlled trials to confirm these results, establish optimal dosing protocols, and explore the broader applicability of allopurinol in clinical practice.

## Introduction and background

Contrast-induced nephropathy (CIN) is a critical clinical concern characterized by acute deterioration in renal function following the administration of contrast media, commonly associated with angiographic procedures. The diagnostic criteria for CIN include either a 25% increase in serum creatinine levels or an absolute rise of 0.5 mg/dL within 48 to 72 hours after contrast exposure, in the absence of alternative causative factors [1]. Acute coronary syndrome (ACS) patients are particularly predisposed to CIN due to frequent hemodynamic instability and the limited time available for prophylactic hydration, which contributes to prolonged hospital stays and elevated mortality rates [2]. Additionally, individuals with diabetes, chronic kidney disease, or heart failure face an elevated risk of developing CIN.

As the utilization of contrast-dependent imaging and percutaneous interventions continues to expand, the prevalence of CIN has concurrently increased, affecting up to 50% of high-risk patient populations. This alarming incidence has prompted healthcare providers to prioritize risk-reduction strategies, including the implementation of hydration protocols, the use of alternative contrast agents, and the exploration of pharmacological interventions such as allopurinol. In response to these challenges, ongoing research is dedicated to enhancing early detection and management approaches, with the overarching goal of safeguarding vulnerable populations and optimizing patient outcomes in the context of contrast-induced complications [3].

## Review

Acute coronary syndrome (ACS) represents a subset of cardiovascular diseases and is distinct from coronary heart disease (CHD) and coronary artery disease (CAD), both of which are caused by atherosclerosis in the coronary arteries. While CAD can remain asymptomatic, ACS is characterized by clinical symptoms such as unstable angina and myocardial infarction (MI). Mortality rates in ACS, particularly among individuals under the age of 70, are higher in men compared to women. In 2020, age-standardized mortality rates (ASMRs) were highest in low-income regions. Conversely, substantial reductions in ACS mortality over the past two decades have been observed in Europe and North America. However, regions such as Asia, Latin America, and Africa have shown stable or increasing mortality rates, underscoring the need for region-specific strategies to address this growing burden. The slower progress in low-income nations reflects the significant impact of socioeconomic factors on healthcare outcomes [4-6].

Coronary artery disease remains a leading cause of mortality globally and is often treated using percutaneous coronary intervention (PCI). This minimally invasive procedure is designed to restore arterial patency by employing techniques such as balloon angioplasty and stent placement, guided by the use of contrast material for arterial visualization. Although PCI is effective, it is associated with potential complications, including coronary artery dissection, bleeding, renal failure, stroke, and myocardial infarction. These risks are heightened in patients with advanced age, female gender, renal dysfunction, diabetes, or a low body mass index (BMI) [7, 8].

Contrast-induced nephropathy (CIN) is a particularly concerning complication of PCI, especially in patients with ACS. The risk of CIN is threefold higher in this population, particularly among those with pre-existing diabetes or renal dysfunction, where incidence rates can reach as high as 50%. Even in individuals with normal or slightly elevated baseline creatinine levels, CIN has an incidence of up to 13.9%, contributing to increased mortality, the need for renal replacement therapy, and recurrent myocardial infarction. These findings emphasize the importance of effective risk assessment and the implementation of preventive strategies [9].

CIN, defined as an acute decline in kidney function following the administration of iodinated contrast agents, presents significant challenges for early detection. Traditional methods relying on serum creatinine measurements have limitations in identifying CIN at its early stages. Emerging biomarkers such as plasma cystatin-C (Cys-C), neutrophil gelatinase-associated lipocalin (NGAL), and urinary liver-type fatty acid-binding protein (L-FABP) have demonstrated potential in enhancing early detection and risk prediction in patients undergoing PCI [10-13].

The development of CIN is strongly associated with pre-existing renal dysfunction, with chronic kidney disease (CKD) being the most significant risk factor. Up to 60% of CIN cases occur in patients with CKD, and approximately 56% of these patients may progress to irreversible renal failure. Other contributing risk factors include advanced age, female gender, heart failure, dehydration, cirrhosis, and atherosclerosis. The risk is also influenced by the volume and type of contrast media used during coronary angiography, with larger volumes correlating with increased CIN and mortality rates. However, the absence of CKD or diabetes is associated with a significantly lower risk of CIN [14-16].

Contrast media are categorized based on their osmolality and ionic properties, which influence their toxicity profile. High-osmolar contrast media (HOCM) carry greater risks of adverse reactions, including renal toxicity, while low-osmolar contrast media (LOCM) and iso-osmolar contrast media (IOCM), such as iodixanol (Visipaque®), are associated with improved safety profiles [17-19].

The pathogenesis of CIN involves several mechanisms, including renal medullary hypoxia, tubular obstruction caused by crystal formation, and direct tubular injury mediated by the production of reactive oxygen species (ROS). Elevated uric acid levels, or hyperuricemia, further exacerbate the risk of CIN in patients with CKD undergoing coronary angiography [14, 20, 21].

Preventive strategies for CIN focus on identifying high-risk patients and implementing effective interventions. Adequate hydration, particularly through intravenous isotonic saline administration, remains the most effective measure for mitigating CIN risk. This approach enhances renal protection by facilitating the clearance of contrast agents and modulating vasoconstriction through the regulation of the renin-angiotensin system [10, 22-26]. Although sodium bicarbonate has been explored for its potential benefits in alkalinizing urine and reducing oxidative stress, isotonic saline remains the preferred method [25, 26].

Antioxidants, including sodium bicarbonate, N-acetylcysteine (NAC), and ascorbic acid, have demonstrated promise in reducing oxidative stress, a key factor in CIN development [18, 25]. Statins have also shown efficacy in lowering the risk of CIN by improving endothelial function, reducing oxidative stress, and attenuating inflammation, making them particularly beneficial for high-risk PCI patients [27].

Allopurinol, a xanthine oxidase inhibitor, has emerged as a potential agent for CIN prevention. By reducing serum uric acid levels and inhibiting ROS production, allopurinol addresses key mechanisms underlying CIN pathogenesis. Although existing studies report mixed findings, meta-analyses suggest that allopurinol may be effective in high-risk patients, particularly when combined with other preventive strategies such as hydration. Additionally, allopurinol has shown potential to improve outcomes in patients undergoing PCI for acute ST-elevation myocardial infarction (STEMI) [1, 3, 28-30].

Rationale for the study

The study aims to update and clarify the evidence regarding the use of allopurinol for preventing contrast-induced nephropathy (CIN) in patients undergoing percutaneous coronary intervention (PCI). Despite promising data suggesting that allopurinol may reduce renal complications associated with contrast media, findings from previous research have been inconsistent due to variability in study designs, patient populations, and methodologies. Since the last meta-analysis in 2021, new studies have emerged, warranting a comprehensive synthesis of the latest evidence. This systematic review and meta-analysis seek to evaluate the effectiveness and safety of allopurinol, offering current insights to support clinical decision-making and guide future research. The findings have the potential to influence clinical practice and improve patient outcomes in PCI settings.

Methods

Study Design

This systematic review and meta-analysis were conducted in accordance with the PRISMA (Preferred Reporting Items for Systematic Reviews and Meta-Analyses) guidelines. The study aimed to synthesize evidence on the efficacy of allopurinol in preventing contrast-induced nephropathy (CIN) in patients undergoing percutaneous coronary intervention (PCI).

Search Strategy

A comprehensive literature search was conducted using PubMed (MEDLINE), Google Scholar, and ScienceDirect to identify relevant studies. The search included a combination of Medical Subject Headings (MeSH) terms and keywords such as "contrast-induced injury," "acute kidney injury," "allopurinol," "percutaneous coronary intervention," "contrast medium," and "coronary angiography." Due to access restrictions, results from the Cochrane database were excluded. The search was limited to randomized controlled trials (RCTs) published in English.

Eligibility Criteria

The inclusion and exclusion criteria were defined using the Population, Intervention, Comparison, and Outcomes (PICO) framework, an appropriate tool for this type of research (Tables 1-2).

**Table 1:** Inclusion criteria

Criteria	Details
Participants	Adult patients undergoing percutaneous coronary intervention with various contrast media.
Intervention	Administration of allopurinol.
Comparators	Controls not receiving allopurinol.
Outcome	Studies reporting renal outcomes.
Study Design	Randomized controlled trials (RCTs) published in English.

**Table 2:** Exclusion criteria

Criteria	Details
Participants	Adult patients not undergoing percutaneous coronary intervention.
Intervention	Absence of allopurinol administration.
Comparators	Not applicable.
Outcome	Studies not reporting renal outcomes.
Study Design	Non-randomized controlled studies and studies not specifically including percutaneous coronary intervention patients.

Outcomes of Interest

The primary outcome was the incidence of CIN, defined as an increase in serum creatinine (SCr) levels of ≥0.5 mg/dL or ≥25% above baseline within 48 hours post-contrast administration. Secondary outcomes included changes in other renal function parameters, clinical outcomes, and adverse events.

Study Selection and Data Extraction

The study selection process involved two stages. First, all identified articles were screened for relevance, and duplicates were removed using reference management software. In the second stage, potentially eligible studies were assessed based on predefined inclusion and exclusion criteria.

Data extraction was performed independently by two reviewers to ensure accuracy and reliability. Extracted data included study design, participant characteristics, details of the intervention, comparator information, and reported outcomes. Any discrepancies between reviewers were resolved through consensus or, if necessary, consultation with a third reviewer.

Statistical Analysis

A meta-analysis was performed using a random-effects model to account for potential heterogeneity among studies. Effect sizes were expressed as odds ratios (ORs) or mean differences (MDs) with 95% confidence intervals (CIs). Heterogeneity was assessed using the I² statistic, with significant heterogeneity defined as I² >50%. Sensitivity analyses were conducted to explore sources of heterogeneity when necessary. Visual representation of results was provided through forest plots, and statistical significance was determined at p < 0.05. All statistical analyses were conducted using Review Manager (RevMan, The Cochrane Collaboration, London, UK).

Risk of Bias Assessment

The risk of bias was evaluated independently by two reviewers using the Cochrane Collaboration’s tool. This tool assesses biases across six domains: selection, performance, detection, attrition, reporting, and other biases. Each study was classified as having low, high, or unclear risk of bias in each domain.

Data Management

Data from the included studies were managed using Rayyan, an online reference management software designed to streamline the screening process for systematic reviews (Rayyan Systems Inc., Cambridge, MA). Duplicate and irrelevant entries were excluded during the initial screening phase. Extracted data were verified for accuracy and completeness before statistical analysis. Any conflicts in data interpretation were resolved through group discussion.

Results

Search Results

**Figure 1:**
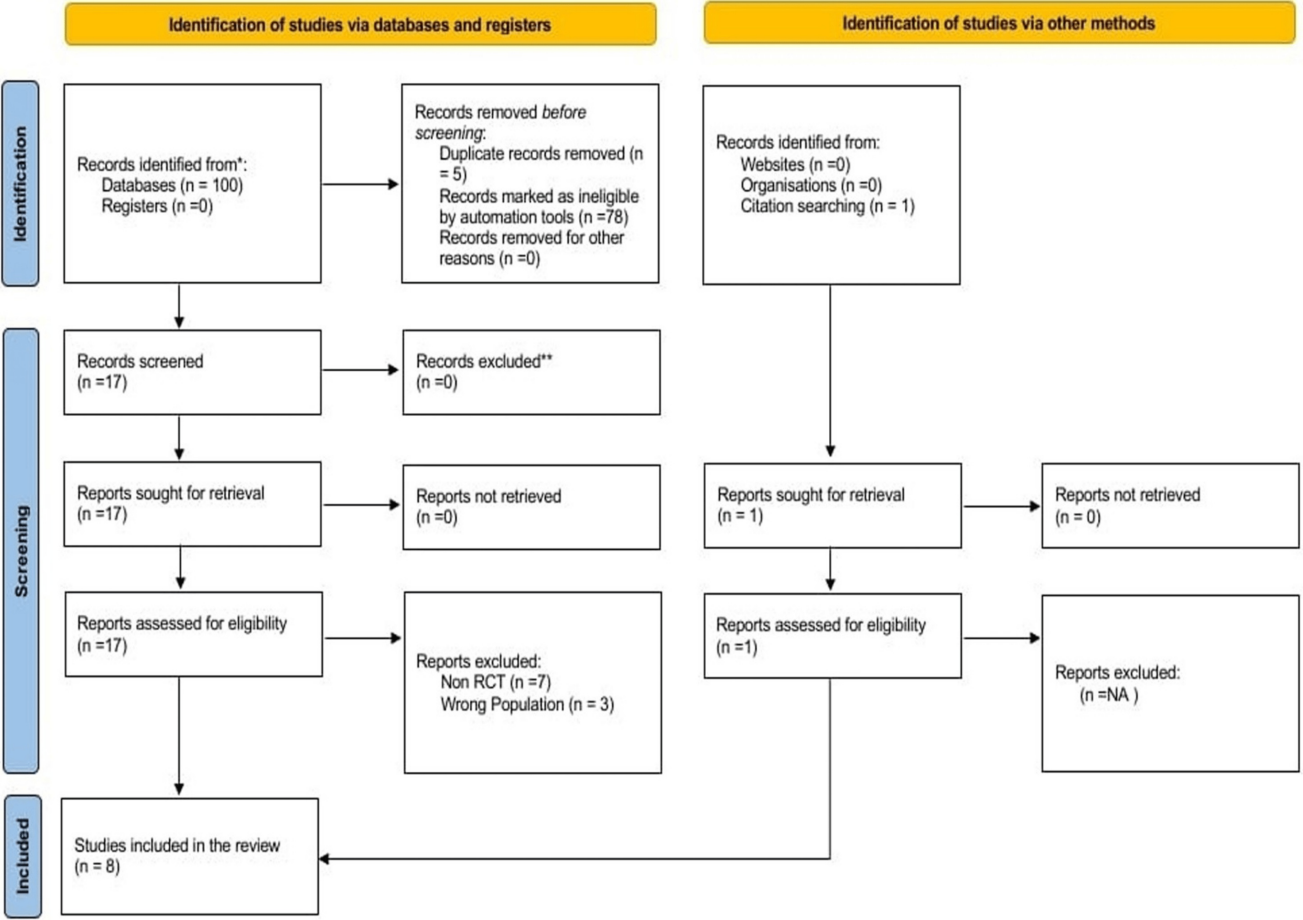
Flowchart depicting the inclusion of studies following the PRISMA guidelines

The systematic review and meta-analysis process is summarized in the PRISMA flowchart (Figure 1). A total of 100 articles were identified through database searches, with an additional article sourced from background research. After removing five duplicates, 96 abstracts were screened, resulting in the exclusion of 78 articles due to irrelevance, such as animal studies or outcomes not aligned with the review objectives. A total of 18 articles underwent full-text review. Of these, seven were excluded because of mismatched populations, and three were excluded due to unsuitable study designs. Ultimately, eight trials met the inclusion criteria and were included in the meta-analysis.

Kumar et al. [31] conducted a prospective trial involving 500 patients who underwent coronary angiography followed by percutaneous coronary intervention (PCI). To ensure clarity and accuracy of results, participants were divided into two distinct groups based on the type of contrast media used: Kumar A, which included patients who received iohexol (Omnipaque), and Kumar B, which included those who received iodixanol (Visipaque). Each group was further subdivided according to three hydration strategies: hydration alone, hydration with N-acetylcysteine (NAC), and hydration with allopurinol. To avoid potential confounding effects, the outcomes for Kumar A and Kumar B were analyzed as two separate studies, as combining results from different contrast media could introduce bias or distort the findings. The inclusion and exclusion criteria applied by the studies under review, along with the definition of contrast-induced nephropathy (CIN), are outlined in detail in Table 3.

**Table 3:** Inclusion and exclusion criteria employed by studies in the meta-analysis MACD: maximum acceptable contrast dose

Authors	Inclusion	Exclusion	Definition of CIN
Erol et al. [1]	Individuals with steady serum creatinine levels of 1.1 mg/dL or higher, who underwent cardiac catheterization or intervention from October 2004 to August 2006.	Patients with acute myocardial infarction (AMI) requiring coronary intervention within 24 hours, cardiogenic shock, acute renal failure, current peritoneal dialysis or haemodialysis, planned post-contrast dialysis, or a history of intravascular contrast administration with expected re-administration within four days.	An increase in baseline serum creatinine concentration by 25%
Kumar et al. (A) and (B) [31]	The Kumar A and Kumar B studies were part of a single prospective trial involving 500 patients who underwent coronary angiography, followed by PCI. Patients were divided into two groups based on the type of contrast media used: Kumar A (iohexol) and Kumar B (iodixanol). Patients eligible for both studies were those consenting to undergo coronary angiography and PCI, regardless of cardiovascular risk factors. They had to receive a contrast dye dose within the maximum permissible limit, calculated as 5 x body weight (kg) / serum creatinine (mg/dL). Angiography-positive patients (with significant coronary artery disease) proceeded to PCI. Patients with contrast-induced nephropathy (CIN) were included if their serum creatinine returned to normal before PCI.	Exclusion criteria for both groups included patients receiving a contrast dye dose exceeding the permissible limit, those taking nephrotoxic medications, and patients with gout or serum uric acid levels >10 mg/dL. Patients with hypersensitivity to allopurinol, CHF or LVEF <40%, unresolved CIN, or an inability to provide informed consent were excluded. Angiography-negative patients (without significant coronary disease) were discharged and excluded from PCI.	A rise in serum creatinine levels of 0.5 mg/dl, or a 25% increase from baseline
Khan et al. [30]	eGFR > 60 ml/min	Individuals with acute coronary syndrome, prior CABG or PPCI, a history of allopurinol use, gout, serum creatinine above 3 mg/dL, eGFR below 60 ml/min, hepatic failure, those on 6-mercaptopurine, warfarin, or azathioprine, and those with an allopurinol allergy were excluded.	A 25% increase in serum cystatin C relative to the patient’s baseline value in the first 24 h
Sadineni et al. [32]	Individuals aged 30 years or older with a consistent serum creatinine level of 1.2 mg/dL or higher were included in the study.	The study excluded individuals with acute renal failure, end-stage renal disease requiring dialysis, recent intravascular contrast administration within 6 days, pregnant or lactating individuals, those undergoing emergent coronary angiography, with a history of contrast media hypersensitivity, in cardiogenic shock, experiencing pulmonary edema, requiring mechanical ventilation, using parenteral diuretics, recent use of N-acetylcysteine, ascorbic acid, or metformin/NSAIDs within 48 hours of the procedure.	A rise in serum creatinine compared to the initial level by either 25% or more, or an absolute increase of 0.3 or greater.
Iranirad et al. [33]	Patients with congestive heart failure, hypertension, and diabetes mellitus, individuals aged 75 years or older, and those with renal insufficiency defined as an estimated glomerular filtration rate (eGFR) less than 60 ml/min/1.73 m² or a baseline serum creatinine level greater than 1.5 mg/dL were considered in the study.	The study excluded patients with end-stage renal insufficiency (eGFR < 15 ml/min), acute renal insufficiency, pregnant or lactating individuals, those with pulmonary edema, cardiogenic shock, multiple myeloma, a history of allergic reactions to contrast agents or allopurinol, recent contrast media exposure within 7 days, uremia, renal failure requiring dialysis, and those using N-acetylcysteine, metformin, dopamine, theophylline, sodium bicarbonate, mannitol, fenoldopam, diuretics, or nephrotoxic medications within 48 hours before the procedure.	A rise in serum creatinine (SCr) levels by 44.2 µmol/l (equivalent to 0.5 mg/dL) or an increase of 25% above the baseline within 24–48 hours.
Bodagh et al. [34]	The study included patients aged over 55 years, those undergoing angioplasty for the first time, individuals undergoing elective procedures, and those with a serum creatinine (SCr) value exceeding 1.1 mg/kg.	The exclusion criteria included a history of acute or chronic renal failure, diabetes mellitus (DM), emergency angioplasty, and a family history of renal diseases.	The study encompassed individuals who were aged 55 years or older, undergoing angioplasty for the first time, participating in elective procedures, and having a serum creatinine (SCr) value surpassing 1.1 mg/kg.
Sultan et al. [35]	Individuals between the ages of 18 and 75 years of any gender, with an estimated glomerular filtration rate (eGFR) less than 60 ml/min/1.73m² (calculated by the CKD-EPI Formula), and those undergoing Percutaneous Coronary Intervention were considered in the study.	The study excluded individuals with acute kidney injury, chronic kidney disease (eGFR < 30 ml/min/1.73m²), renal transplant patients, those in shock, individuals with known contrast agent allergies or recent contrast use (within 7 days), pregnant or lactating individuals, those with sepsis, advanced liver disease, or taking medications like azathioprine, cyclosporine, cyclophosphamide, warfarin, ampicillin, amoxicillin. It also excluded individuals using nephroprotective drugs (e.g., N-acetylcysteine, theophylline, sodium bicarbonate), patients with gout or serum uric acid levels >10 mg/dL, haemoglobin <11 g/dL, those on ACE inhibitors/ARBs, metformin, NSAIDs, diagnosed with malignancy, allergic to or already using allopurinol, and those who refused to participate. Patients exceeding the MACD (5 × weight in kg/serum creatinine) were also excluded.	Contrast-induced nephropathy (CIN) was evaluated in all patients, with the definition being an absolute increase in serum creatinine level of greater than 0.5 mg/dl or a relative increase of more than 25% from the baseline at 48 hours after contrast exposure. This assessment was then compared in both treatment groups.

Baseline Study Characteristics

This meta-analysis included eight randomized controlled trials (RCTs) published up to November 2023, with a total of 929 participants and an average age of 63 years. These studies, conducted by Erol et al. [1], Kumar et al. with its two parts (A and B) [31], Khan et al. [30], Sadineni et al. [32], Iranirad et al. [33], Bodagh et al. [34], and Sultan et al. [35], evaluated the role of allopurinol in preventing contrast-induced nephropathy (CIN) in patients undergoing percutaneous coronary intervention (PCI) and other contrast-based procedures.

Across the trials, the average baseline serum creatinine (SCr) level was 1.28 mg/dL. The contrast agents used included Omnipaque and Visipaque. Most studies administered 300 mg of allopurinol with hydration 24 hours before the procedure. However, two studies used higher doses: Khan et al. [30] administered 1200 mg, while Bodagh et al. [34] used 600 mg. The definition of CIN was consistent across most studies, defined as an increase in serum creatinine of ≥0.5 mg/dL or ≥25% above baseline within 48 hours post-procedure. Notably, Khan et al. [30] used a different biomarker, defining CIN based on a 25% increase in serum cystatin-C within 24 hours post-procedure.

Detailed study characteristics, including participant demographics, interventions, and outcome measures, are provided in the appendix.

Risk of Bias 

The risk of bias graphs and summaries demonstrate a mixed risk profile among the included studies. Concerns regarding selection bias, as evidenced by issues with allocation concealment, and performance bias, due to the lack of blinding of participants and personnel, were notable. These biases may influence the outcomes and interpretations of individual trial results.

The funnel plot analysis, used to assess publication bias, indicates a symmetrical distribution of studies, suggesting minimal publication bias and supporting the reliability of the meta-analysis findings. However, given the inherent limitations of funnel plots, further statistical tests such as Egger's test could provide a more robust assessment of publication bias.

**Figure 2:**
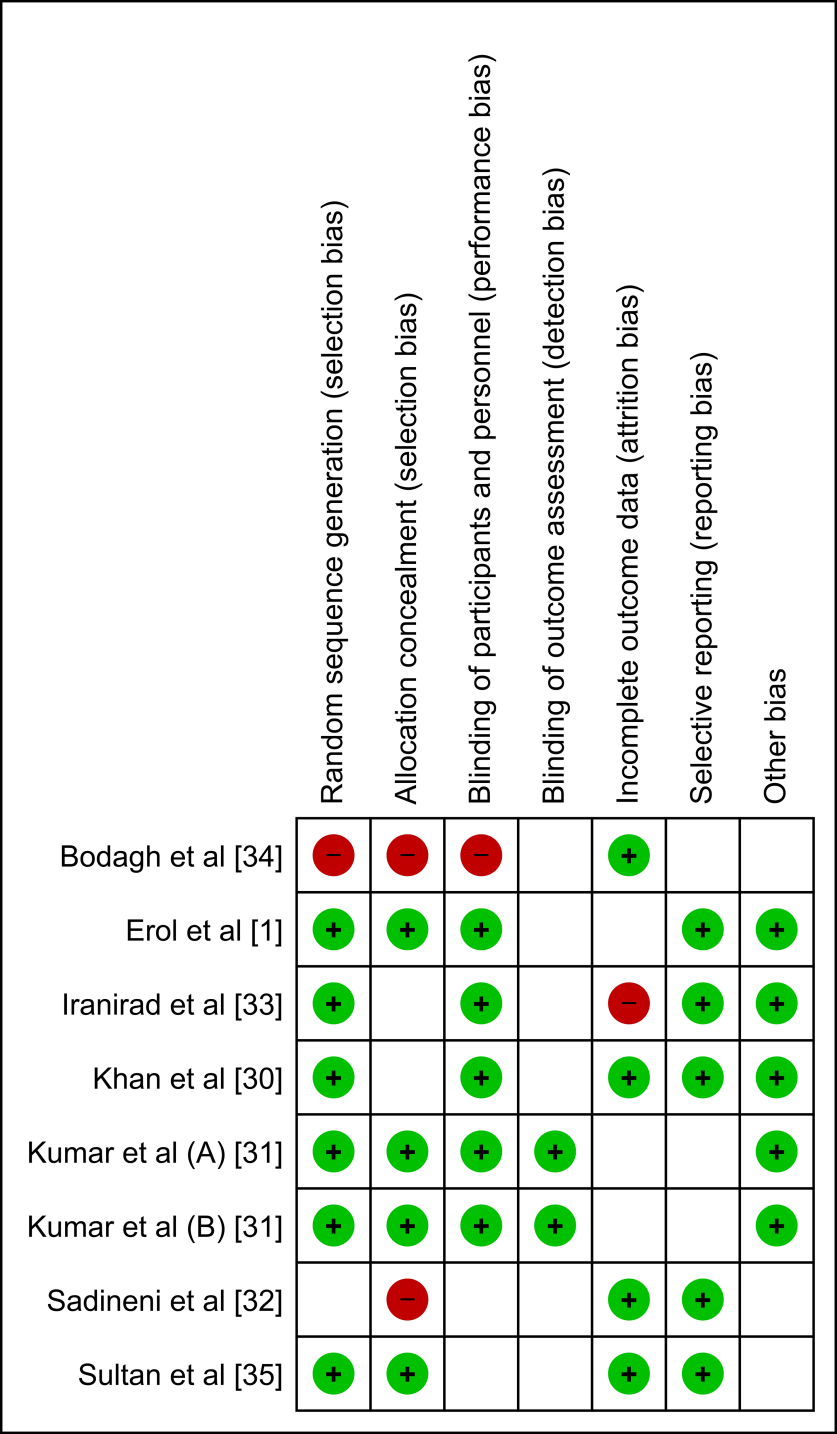
Bias Assessment Indicator for Studies Included in the Meta-Analysis.

**Figure 3:**
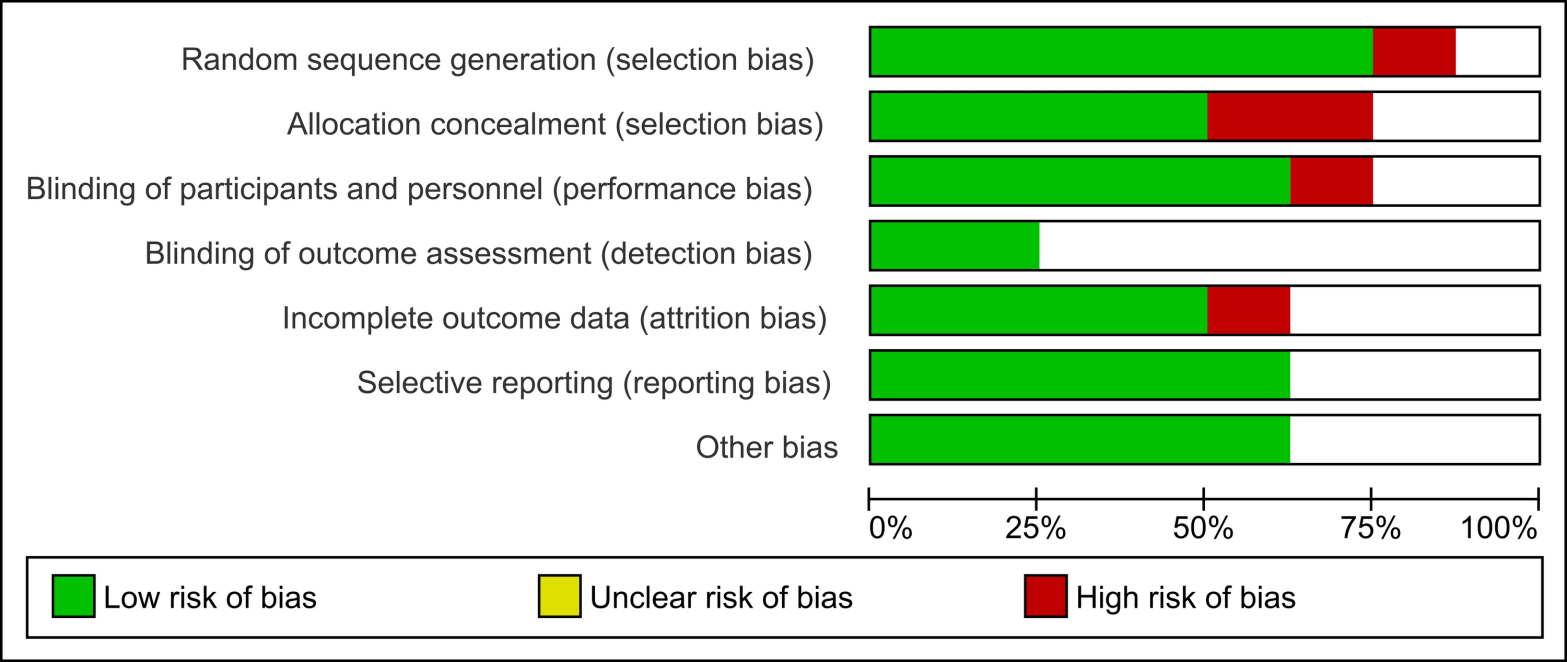
Risk of bias graph for Studies Included in the Meta-Analysis

**Figure 4:**
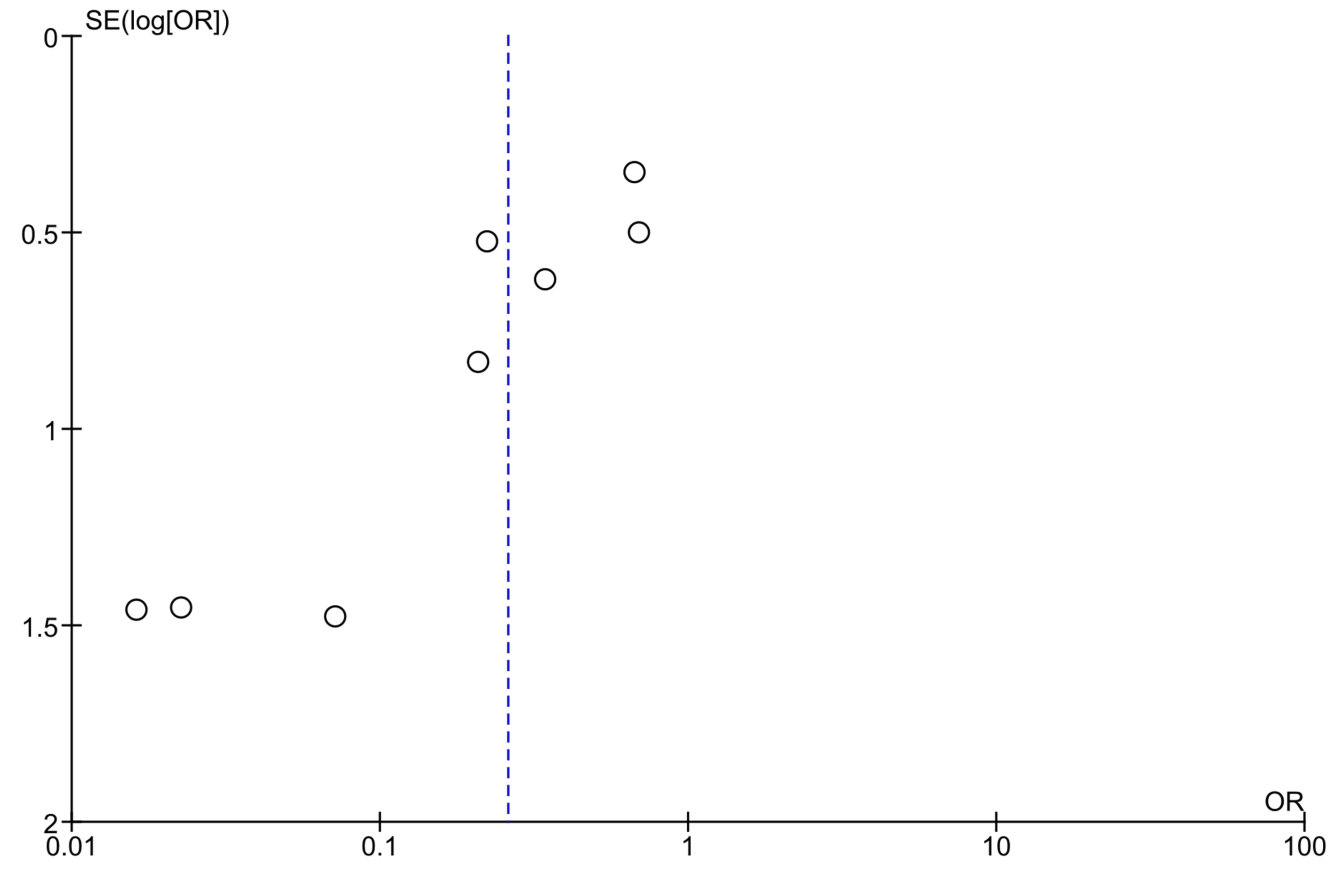
Funnel plot for publication bias in meta-analysis of allopurinol vs. hydration for CIN prevention CIN: contrast-induced nephropathy

Details of the risk of bias and publication bias assessments are presented in Figures 2-4.

Heterogeneity Assessment

**Figure 5:**
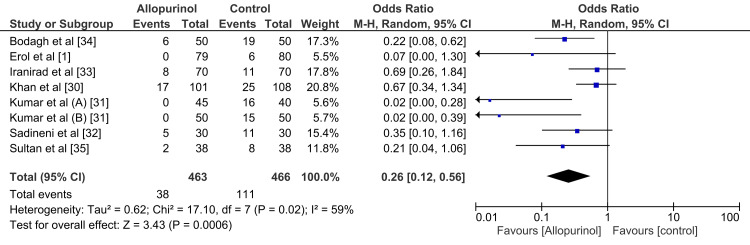
Forest plot of comparison of allopurinol versus hydration in the prevention of contrast-induced nephropathy

In evaluating heterogeneity, the meta-analysis integrated data from several randomized controlled trials investigating the efficacy of allopurinol in preventing contrast-induced nephropathy (CIN). Forest plot analysis revealed a pooled odds ratio of 0.26 [95% CI (0.12, 0.56), P = 0.0006], demonstrating a significant reduction in CIN incidence with allopurinol compared to the control group. While this substantial effect was consistently observed across studies, a moderate-to-high level of heterogeneity was identified (I² = 59%) (Figure 5).

The observed variability likely stems from differences in study designs, patient populations, and procedural details, underscoring the need for cautious interpretation of the pooled results. Individual patient assessments and clinical contexts must be considered when applying these findings in practice. Furthermore, the recognized risk of bias in some studies highlights the importance of future research incorporating rigorous methods, such as improved blinding and allocation concealment, to minimize potential biases and strengthen the reliability of evidence.

Outcomes

The primary outcome of this meta-analysis was the occurrence of contrast-induced nephropathy (CIN), which was defined as an increase in serum creatinine (SCr) levels by either ≥0.5 mg/dL or ≥25% from baseline within 48 to 72 hours after exposure to contrast media. The characteristics and outcomes of the studies included in this meta-analysis are presented in Table 4. 

**Table 4:** Study characteristics and outcomes RCT: randomized controlled trial; CIN: contrast-induced nephropathy

Author	Year	Design	Number of cases			CIN		
			Total	Allopurinol group	Control group	Allopurinol group	Control group	p-value
Erol et al. [1]	2013	RCT	159	79	80	0	6	0.013
Kumar et al. (A) [31]	2014	RCT	85	45	40	0	16	< 0.05
Kumar et al. (B) [31]	2014	RCT	100	50	50	0	15	< 0.05
Khan et al. [30]	2017	RCT	209	101	108	17	25	0.225
Sadineni et al. [32]	2017	RCT	60	30	30	5	11	> 0.05
Iranirad et al. [33]	2017	RCT	140	70	70	8	11	0.459
Bodagh et al. [34]	2019	RCT	100	50	50	6	19	0.003
Sultan et al. [35]	2023	RCT	76	38	38	2	8	0.042

The pooled analysis, as illustrated in Figure 5, revealed a significant reduction in the incidence of contrast-induced nephropathy (CIN) with allopurinol use compared to the control group, yielding an odds ratio of 0.26 (95% CI: 0.12 to 0.56, P = 0.0006).

The study weights contributing to the pooled estimate varied, with the largest contribution from Khan et al., 2017 (20.8%), followed by Iranirad et al., 2017 (17.8%), and Bodagh et al., 2019 (17.3%). Kumar et al., analyzed in two parts (A and B), together contributed 11.3% and demonstrated the lowest odds ratios of 0.02 [95% CI (0.00, 0.28)] and 0.02 [95% CI (0.00, 0.39)], respectively. The pooled results consistently favored allopurinol across studies, with moderate heterogeneity (I² = 59%), likely due to differences in study design, patient populations, and intervention protocols.

Notable findings included significant protective effects of allopurinol in reducing CIN incidence, even in high-risk populations. However, secondary outcomes, such as changes in renal function parameters and adverse events, were inconsistently reported across studies and therefore not included in the pooled analysis.

These findings highlight the robust protective effect of allopurinol against CIN and underline the need for further research to address heterogeneity and explore optimal dosing regimens.

Sensitivity Analysis

**Figure 6:**
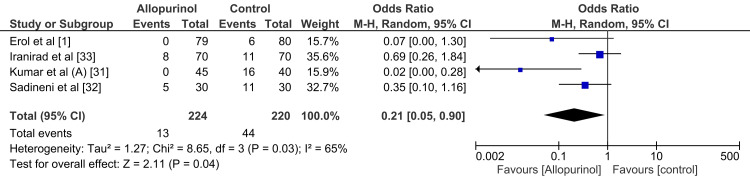
Forest plot of comparison of allopurinol in the prevention of contrast-induced nephropathy in the studies which used Omnipaque contrast agent

The author conducted a sensitivity analysis to examine whether the type of contrast used during the procedure might have influenced the analysis. Consequently, a separate forest plot was generated for studies that used Omnipaque, and the results were compared with those for the entire set of studies. The forest plot analysis revealed a pooled odds ratio of 0.26 [95% CI (0.12, 0.56), P = 0.0006] in the overall studies. In comparison, it demonstrated an odds ratio of 0.21 [95% CI (0.05, 0.90), P = 0.04] in the homogeneous subgroup that used Omnipaque contrast media (Figure 6).

The sensitivity analysis in this study underscores the robustness of allopurinol's protective effect against contrast-induced nephropathy (CIN), with a consistent pooled odds ratio of 0.26 across studies, indicating a substantial reduction in CIN risk. A subgroup analysis of studies using Omnipaque contrast media showed a similar protective effect (pooled odds ratio of 0.21), suggesting that the type of contrast agent does not significantly impact allopurinol’s efficacy. These findings support allopurinol’s broad potential as a CIN preventive measure across various clinical settings and contrast agents. However, more extensive trials are needed to validate these results and examine specific patient contexts.

Discussion 

Against the backdrop of the increasing incidence of contrast-induced nephropathy (CIN), especially within high-risk populations undergoing diagnostic and therapeutic procedures dependent on contrast agents, the results of this study propose a potential protective role for allopurinol in mitigating renal impairment. This finding holds substantial implications for clinical practice, suggesting that the inclusion of allopurinol in prophylactic strategies for CIN could be particularly beneficial, especially in patients diagnosed with acute coronary syndrome (ACS).

The growing recognition of CIN underscores the relevance of these findings as a significant complication associated with contrast-enhanced procedures, especially in vulnerable patient groups. Patients undergoing diagnostic and interventional procedures, such as those with ACS, often face an increased risk of CIN due to the necessity of contrast media. Allopurinol, with its observed potential to reduce the incidence of CIN, emerges as a promising candidate for incorporation into preventive measures.

Considering the potential protective effects of allopurinol, clinicians managing patients at risk of CIN, especially those with ACS, may contemplate the incorporation of allopurinol into their preventive strategies. This could involve a careful assessment of individual patient profiles, weighing the potential benefits against any contraindications or side effects associated with allopurinol use. As part of a comprehensive approach to minimize the risk of CIN, allopurinol could contribute to improved patient outcomes and a more tailored, patient-centered approach to medical interventions involving contrast media. Further research and clinical trials are warranted to validate and refine these findings, establishing a more evidence-based framework for the integration of allopurinol in CIN prevention strategies.

In our comprehensive meta-analysis of randomized controlled trials exploring the efficacy of allopurinol in preventing contrast-induced nephropathy (CIN), a notable and statistically significant reduction in CIN incidence was observed (pooled odds ratio: 0.26 [95% CI (0.12, 0.56), P = 0.0006]). This protective effect remained consistent across diverse studies, despite a moderate level of heterogeneity (I² = 59%), which may be attributed to variations in study designs, patient populations, or procedural details.

Comparing our findings with the study conducted by Ma et al., encompassing eight randomized controlled trials with 1,141 patients, both investigations demonstrated a reduced risk of contrast-induced acute kidney injury (CI-AKI) associated with allopurinol (relative risk (RR) 0.39, 95% CI 0.20, 0.74, P = 0.004) [36].

Furthermore, in alignment with the analysis by Xin et al., which evaluated five randomized controlled trials involving 754 patients undergoing percutaneous coronary intervention with preprocedural hydration, our study indicated a significant reduction in CI-AKI incidence with allopurinol (risk ratio: 0.37, p = 0.01) [37]

Additionally, when compared with the findings of Bellos et al., who included six studies with a total of 918 patients, our results were consistent, demonstrating a significantly reduced incidence of contrast-induced nephropathy with allopurinol compared to hydration alone (odds ratio: 0.29, 95% CI: 0.09-0.90) [38].

The coherence of these results across multiple studies provides compelling support for the potential protective role of allopurinol against CIN, reinforcing its consideration as a valuable component of preventive strategies for patients undergoing percutaneous coronary intervention.

However, the observed heterogeneity among the studies underscores the importance of a cautious interpretation of the pooled results. This emphasizes the need for individual patient assessment and consideration of the specific clinical context when applying these findings. Additionally, the identified risk of bias in the studies calls for future research to adopt more rigorous blinding and allocation concealment methods, aiming to minimize potential biases and enhance the robustness of the evidence.

## Conclusions

This meta-analysis presents compelling evidence supporting the efficacy of allopurinol in reducing the risk of contrast-induced nephropathy (CIN), particularly in high-risk populations. The findings demonstrate a significant reduction in CIN incidence; however, moderate heterogeneity and methodological limitations in the included studies warrant cautious interpretation. Variability in study designs, patient populations, and intervention protocols underscores the necessity for further robust, well-designed, and adequately powered randomized controlled trials to validate these findings.

Should subsequent research confirm allopurinol's protective role, it may represent an important advancement in CIN prevention strategies. Its established safety profile, affordability, and dual mechanism of reducing oxidative stress and serum uric acid make it a promising candidate for integration into clinical practice.

In conclusion, while this analysis highlights the potential of allopurinol as an adjunctive preventive strategy for CIN, the findings emphasize the need for ongoing rigorous investigations to refine its clinical applications and establish evidence-based guidelines for its use.

## Appendices

Table 5 shows the baseline renal characteristics, contrast media details, and allopurinol dosage from the articles included in the study.

**Table 5:** Baseline renal characteristics, contrast media details, and allopurinol dosage from studies in the systematic review and meta-analysis eGFR: estimated glomerular filtration rate; sCr: serum creatinine

Author		Erol et al. [1]	Kumar et al. (A) [31]	Kumar et al. (B) [31]	Khan et al. [30]	Sadineni et al. [32]	Iranirad et al. [33]	Bodagh et al. [34]	Sultan et al. [35]
Year		2013	2014	2014	2017	2017	2017	2019	2023
Design		RCT	RCT	RCT	RCT	RCT	RCT	RCT	RCT
eGFR (allopurinol)	Mean	53.00	83	78	-	-	75.8	-	53.70
	SD	15.00	-	-	-	-	28.1	-	4.70
eGFR (control)	Mean	51.00	83	78	-	-	74.2	-	54.80
	SD	16.00	-	-	-	-	24.9	-	3.80
sCr (allopurinol) - mg/dl	Mean	1.43	1	1.1	0.88	1.91	1.2	-	1.31
	SD		-	-	0.24	0.72	0.3	-	0.07
sCr (control) - mg/dl	Mean	1.48	1	1.1	0.96	2.19	1.1	-	1.28
	SD		-	-	0.24	1.01	0.3	-	0.09
Contrast agent		Omnipaque	Omnipaque	Visipaque	-	Omnipaque	Omnipaque	-	non-ionic contrast
Contrast volume (allopurinol) - ml	Mean	121	-	-	212.3	68.7	40.1	-	146.8
	SD	25	-	-	84.1	46.77	15.5	-	10.7
Contrast volume (control) - ml	Mean	119	-	-	207.7	77.33	40.1	-	148.4
	SD	26	-	-	76.5	43.3	14.9	-	9.8
Allopurinol dose in mg		300	300	300	1200	300	300	600	300

Table 6 shows the baseline characteristics and demographic data of the studies included in the systematic review and meta-analysis.

**Table 6:** Baseline characteristics and demographic data of the studies incorporated in this systematic review and meta-analysis DM: diabetes mellitus; HTN: hypertension, EF: ejection fraction

Author		Erol et al [1]	Kumar et al. (A) [31]	Kumar et al. (B) [31]	Khan et al. [30]	Sadineni et al. [32]	Iranirad et al. [33]	Bodagh et al. [34]	Sultan et al. [35]
Year		2013	2014	2014	2017	2017	2017	2019	2023
Allopurinol mean age	Mean	65	66	68	60.24	62.9	60.3	63.5	57.6
	SD	9			10.1	8.67	12.6	7.4	7.4
Control mean age	Mean	65	66	68	59.31	62.6	62.1	63.8	58.4
	SD	9	-	-	11.7	11.84	10.4	7.7	6.5
Patients with DM	Allopurinol	21(25)	-	-	37(36.3)	17	29(41.4)	-	13(34.2)
	Control	22(27)	-	-	36(34.3)	19	27(38.5)	-	13(34.2)
Patients with HTN	Allopurinol	40(50)	-	-	69(67.7)	20	45(64.2)	-	13(34.2)
	Control	47(58)	-	-	65(61.3)	-	42(60.0)	-	15(39.5)
Patients who smoke	Allopurinol	20(25)	-	-	15(14.7)	-	23(32.8)	-	-
	Control	21(25)	-	-	23(21.7)	-	16(22.8)	-	-
EF allopurinol group	Mean	51	-	-	-	-	40.6	-	-
	SD	11	-	-	-	-	9.7	-	-
EF control group	Mean	53	-	-	-	-	41.8	-	-
	SD	9	-	-	-	-	9.2	-	-
